# Diversity and Plasticity of Th Cell Types Predicted from Regulatory Network Modelling

**DOI:** 10.1371/journal.pcbi.1000912

**Published:** 2010-09-02

**Authors:** Aurélien Naldi, Jorge Carneiro, Claudine Chaouiya, Denis Thieffry

**Affiliations:** 1 Technological Advances for Genomics and Clinics, INSERM U928, Marseille, France; 2 Center for Integrative Genomics, University of Lausanne, Lausanne, Switzerland; 3 Instituto Gulbenkian de Ciência, Oeiras, Portugal; 4 CONTRAINTES Project, INRIA Paris-Rocquencourt, Rocquencourt, France; 5 Institute de Biologie de l'Ecole Normale Supérieure, CNRS 8197, INSERM 1024, Paris, France; New York University, United States of America

## Abstract

Alternative cell differentiation pathways are believed to arise from the concerted action of signalling pathways and transcriptional regulatory networks. However, the prediction of mammalian cell differentiation from the knowledge of the presence of specific signals and transcriptional factors is still a daunting challenge. In this respect, the vertebrate hematopoietic system, with its many branching differentiation pathways and cell types, is a compelling case study. In this paper, we propose an integrated, comprehensive model of the regulatory network and signalling pathways controlling Th cell differentiation. As most available data are qualitative, we rely on a logical formalism to perform extensive dynamical analyses. To cope with the size and complexity of the resulting network, we use an original model reduction approach together with a stable state identification algorithm. To assess the effects of heterogeneous environments on Th cell differentiation, we have performed a systematic series of simulations considering various prototypic environments. Consequently, we have identified stable states corresponding to canonical Th1, Th2, Th17 and Treg subtypes, but these were found to coexist with other transient hybrid cell types that co-express combinations of Th1, Th2, Treg and Th17 markers in an environment-dependent fashion. In the process, our logical analysis highlights the nature of these cell types and their relationships with canonical Th subtypes. Finally, our logical model can be used to explore novel differentiation pathways *in silico*.

## Author Summary

T lymphocytes play a key role in the regulation of the immune response in mammals. Various T-helper subtypes (Th1, Th2, Th17, Treg,…) have been identified over the years, characterised by the expression of specific transcription factors and cytokines, which have a critical influence on the selection of different immune responses, driving pro-inflammatory or allergic responses, promoting alternative antibody classes, or preventing (auto)immunity by inhibiting the activation and proliferation of other cells. To gain insight into the heterogeneity and the plasticity of late T-helper lineages, we have built an integrated model of the regulatory network and signalling pathways controlling Th cell differentiation. Relying on a logical modelling framework, we have performed a systematic series of simulations to assess the effects of heterogeneous environments on Th cell differentiation. We have identified stable states corresponding to canonical Th1, Th2, Th17 and Treg subtypes, but also to hybrid cell types co-expressing combinations of Th1, Th2, Treg and Th17 markers in an environment-dependent fashion. Our analysis highlights the nature of these cell types and their relationships with canonical Th subtypes.

## Introduction

Alternative cell differentiation pathways are believed to arise from the concerted action of signalling pathways and transcriptional regulatory networks. However, the prediction of mammalian cell differentiation from the knowledge of the presence of specific signals and transcriptional factors is currently a daunting challenge. In this respect, the vertebrate hematopoietic system, with its many branching differentiation pathways and cell types, is a compelling case study. In particular, numerous publications describe molecular and genetic interactions involved in the control of the late stages of (TCR
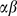
+, CD4+) T helper (Th) cell differentiation, and yet our current knowledge on cell lineage branching is clearly incomplete, as demonstrated by recent reports on novel Th cell types [1]–[4]. The existence of some of these cell types was revealed following the identification of novel transcription factors [2] or cytokines [1], [3]. However, several Th cell phenotypes recently described likely result from reassorted expression of already known genes [4], [5].

Beyond the expression of diverse and cell-specific antigen receptor genes, the appreciation of the heterogeneity of late Th cell lineages emerged from the characterisation of Th1 and Th2 cell types [6]. Both cell types arise following the sustained activation of uncommitted Th0 cell precursors and can be characterised by the expression of mutually exclusive sets of cytokines: Th1 cells produce IFN-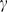, whereas Th2 cells express IL-4, IL-5 and IL-6. These cytokine profiles have a critical influence on the selection of a specific immune response, driving pro-inflammatory or allergic responses, and promoting alternative antibody classes. The cellular dichotomy has been mechanistically explained by the mutual inhibition between the master transcription factors T-bet and GATA-3 at the single cell level, as well as by cross-regulatory mechanisms at the cell population level [7]. Indeed, the cytokines produced by each Th subtype drives the differentiation of precursors into the same pathway while inhibiting alternative pathways.

Additional T-helper subtypes have been recently identified. Regulatory T cells that depend on the transcription factor Foxp3 are capable of preventing (auto)immunity by inhibiting the activation and proliferation of other cells [8]. Furthermore, pro-inflammatory Th17 cells expressing IL-17 and dependent on ROR
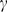
t have been characterised. Current evidences indicate that late Th cell differentiation pathways are more complex and likely comprise further, non-canonical cell types [5], [9]–[12], whose mechanistic underpinnings and functional roles remain to be established.

Effective immunity to many fungi and bacteria requires that the T cell response is dominated by pro-inflammatory effector Th1 or Th17 cells. Allergic reactions, whether beneficial or deleterious, are strictly dependent on Th2 cells. Avoiding spontaneous autoimmunity or controlling the collateral damage of effective immune responses to infection involves a fine balance between regulatory T cells and other Th cells. Genetic defects or accidental failures affecting this delicate balance can lead to irreversible immunopathology.

The issue is not only how heterogeneous are these cell types, but, perhaps more importantly, how is the heterogeneity of Th cells sustained and controlled? How does antigen-specific memory correlate with the predominance of an appropriate Th cell branch? How plastic or resilient are the differentiated Th cells?

During their lifetime, naive and memory Th cells face a changing environment in time and space. The lymphoid tissues are heteregeneous and provide variable local cytokine contexts to circulating Th cells. How robust are the Th subtypes with respect to these heterogeneous environments? Will a Th cell that differentiated into a Th1 phenotype in a lymph node always remain in this state or can it switch to another cell type if it faces a different environment? Evidences for substantial plasticity have been recently reported. For example, *in vitro* stimulation of Th2 cells in the presence of TGF-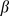 generates a non-canonical cell type expressing IL-10 and IL-9 in the absence of Foxp3 [5], [10]. Furthermore, Foxp3+ regulatory T cells loose the expression of this key transcription factor in the absence of effector T cells *in vivo* and can then drive inflammatory responses [13].

How many instances of such conversions should be expected? Are some Th cells irreversibly committed and others more plastic? It is difficult to address these questions directly due to the current impossibility to follow a single cell as it circulates in the body, during the rather long time scale of cell differentiation. Instead, studies are usually made on cell populations and therefore measure the predominance of one or another cell type. However, using mathematical modelling, these questions can be addressed in terms of stability and robustness of differentiation states of the molecular network underpinning the cellular phenotypes.

Mathematical modelling has been used recurrently to formalise hypothetic regulatory schemes in immunology. Early phenomenological models represented the development of Th1 vs Th2 responses from naive Th0 cells using ordinary differential equations [14]–[17]. In these models, the subcellular molecular network controlling the state of the cell was implicit behind the transitions between cell types. These models accounted for the role of cell interactions in driving population commitment and sustained polarised responses. In general, they featured mutual inhibitions among cell populations, thereby enabling multistability. Alternative population stationary states were interpreted as polarised cell responses. However, such cell population models are unable to predict novel cell types or to question their plasticity because cell properties are hardwired in the model structure.

More recently, models of the cellular networks driving Th cell differentiation and polarisation have been formulated using logical [18] (for an earlier logical model of T-cell regulation, see [19]) or ordinary differential equations [20], [21]. These models assume cross-inhibiting master transcription factors to generate canonical Th subtypes, thereby precluding cell plasticity.

In this paper, we propose an integrated, comprehensive model of the regulatory network and signalling pathways accounting for the core control of Th cell differentiation. As most available data are qualitative, we rely on a qualitative, logical formalism to perform extensive dynamical analyses. To cope with the size and complexity of the resulting network, we use an original model reduction approach described in detail elsewhere [22].

To assess the effects of heterogeneous environments on Th cell differentiation, we have performed systematic simulations, considering various prototypic environments. As we shall see, stable states corresponding to canonical Th1, Th2, Th17 and Treg subtypes are readily identified, but they are found to coexist with other hybrid cell types that co-express combinations of Th1, Th2, Treg and Th17 markers in an environment-dependent fashion. In the process, our logical analysis highlights the nature of these cell types and their relationships to canonical Th subtypes.

## Methods

### Logical modelling formalism

The precise roles of the different molecular species involved in the regulation of T cell differentiation are sparsely known. Even in the cases where direct regulatory interactions have been documented, little or no quantitative information is available on the relative strengths or rates of these processes.

The extended logical formalism [23],[24] is a discrete modelling framework well adapted to biological systems where available information is qualitative. In this framework, a regulatory network is modelled in terms of a *regulatory graph*, where nodes represent regulatory components (proteins, complexes, genes, etc.), whereas arcs represent interactions between these components (*i.e* transcriptional activations or inhibitions, phosphorylations, etc.). In addition, each regulatory component is associated with a logical variable denoting its qualitative concentration or *level of activity*. In many cases, Boolean variables capture the most relevant situations (*i.e.* a Boolean variable takes the value 1 if the component is present or active, 0 otherwise). It is worth noting that components may represent phenomenological features besides specific molecular species (*e.g.* cell proliferation, see Table 1). Whenever needed (*i.e* when different levels of a component have distinct functional consequences), multi-valued variables are introduced. In our Th model, ternary variables have been associated with several interleukin receptor components (IL4RA, IL4R, IL2R, IL12RB1), as well as with STAT5, which can be up-regulated depending on signalling.

**Table 1:** List of regulatory components.

Component(s)	Qualification	Behaviour	Reference(s)
IFNG_e, TGFB_e, IL{2,4,6,10,12,15}_e, IL{17,21,23,27}_e	External cytokines.	Input of the model representing the external environment. We do not consider the arrest of the activation.	
APC	Denotes the presence of an Antigen-Presenting Cell.	Input of the TCR module. We do not consider the arrest of the activation.	
CGC, IFNGR{1,2}, IL{4,6,10,15,27}RA, GP130, IL{2,10}RB	Subchains of the cytokine receptors.	Assumed to be constitutively expressed at functional levels.	
IL12RB2	Subchain of IL-12R.	Inhibited by STAT6 (present otherwise).	[18], [53]
IL12RB1	Subchain of the IL-12 and IL-23 receptors.	Always present with a higher level (required for IL-12 signalling) in presence of IRF1.	[54]
IL2RA	High affinity subchain of the IL-2 receptor.	Activated by NFAT, NFKB, STAT5, SMAD3 and FOXP3.	[32], [55]
IL4RA	Subchain of the IL-4 receptor.	Constitutively expressed, it is upregulated by a high level of STAT5.	
IFNGR, TGFBR, IL{4,6,10,15,17}R, IL{21,23,27}R	Cytokine receptors, composed of subchains as described in Table 2.	Active when their subchains and the cytokine (external or from the same cell) are present.	
IL12R	IL-12 receptor.	As other receptors but requires a higher level of IL12RB1.	[54]
IL23R	IL-23 receptor.	As other receptors but also requires ROR 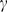 t and STAT3.	[56]
IL4R	IL-4 receptor.	As other receptors, high level of receptor requires a high level of IL4RA.	
IL2R	IL-2 receptor, composed of three subchains (CGC, IL-2R 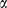 and IL-2R 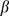 ).	CGC and IL2RB are mandatory, while IL2RA is only needed for higher levels of IL2R.	[32]
TCR, CD28	T Cell Receptor and its co-receptor.	Activated by APC.	
IKB	Denotes I 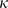 B.	Inhibited by the TCR pathway.	[30]
NFKB	Denotes NF 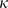 B.	Inhibited by IKB and FOXP3.	[57]
IRF1	Transcription factor.	Activated by STAT1.	[54]
STAT1	Transcription factor.	Activated by IFNBR, IFNGR and IL27R.	[18], [58], [59]
STAT3	Transcription factor.	Activated by IL6R, IL10R, IL21R, IL23R, and IL27R.	[34], [59]
STAT4	Transcription factor.	Activated by IL12R and inhibited by GATA3.	[18]
STAT5	Transcription factor.	Activated by IL2R, IL4R, and IL15R. High levels of IL2R or IL4R are required for high levels of STAT5.	[32]
STAT6	Transcription factor.	Activated by IL4R.	[18]
proliferation	Denotes cell proliferation.	Triggered by high levels of STAT5, its arrest is not considered here. We assume that cell proliferation is required for the production of all cytokines but IL2.	[60], [61]
NFAT	Transcription factor.	Activated by TCR and CD28. We assume it is required for the production of all cytokines.	[32], [62], [63]
TBET	Denote T-bet, the master switch for the Th1 subtype.	Activated by itself and STAT1 and inhibited by GATA3.	[18]
RUNX3	Transcription factor	Activated by TBET.	[64]
GATA3	Denotes GATA-3, the master switch for the Th2 sub type.	Activated by itself and STAT6 and inhibited by TBET.	[18]
FOXP3	Transcription factor specific to Treg cells.	Activated by NFAT, TGFB (through SMAD3), and IL2 (through STAT5) and inhibited by IL6 (through STAT3). Based on promoter binding data, we further assume inhibition by STAT1 and RORGT.	[37], [55], [65]–[69]
RORGT	Denotes ROR 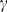 t, required for the production of IL17.	Self-maintained and activated by STAT3 and TGFBR. Potential intermediate in STAT3 activation by TGFBR.	[11], [43], [56], [70]
TGFB	Denotes TGF- 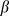 .	Produced by the Treg, assumed to be activated by FOXP3.	
SMAD3	Signal transduction component.	Activated by TGFB.	[55], [67]
IFNG	Denotes IFN- 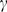 .	Activated by NFAT, proliferation, TBET/RUNX3 and STAT4/IRAK. Activation by NFAT inhibited by FOXP3. Inhibited by STAT3.	[18], [57], [64]
IL2	Denotes interleukin-2.	Activated by NFAT anf NFKB. STAT5 and STAT6 cooperate to inhibit IL2 production. FOXP3 cooperates with NFAT to inhibit IL2. TBET cooperates with RelA (NF 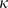 B subunit) to inhibit IL2.	[32], [68], [71], [72]
IL4	Denotes interleukin-4.	Activated by GATA3, NFAT and proliferation. TBET and RUNX3 inhibit IL4 cooperatively. FOXP3 blocks its activation by NFAT. STAT1 inhibits IL4 through IRF1.	[18], [57], [64], [73], [74]
IL10	Denotes interleukin-10.	Activated by NFAT, proliferation, GATA3 IL6 and TGFBR (probably through STAT3).	[34], [75]
IL17	Denotes interleukin-17.	Activated (cooperatively) by STAT3 and RORGT and inhibited by IL2 (through STAT5) and FOXP3. We further assume inhibitions by STAT1 and STAT6.	[11], [43], [56], [59], [76], [77]
IL21	Denotes interleukin-21.	Activated by STAT3.	[43]
IL23	Denotes interleukin-23.	Activated by STAT3.	[43], [56]

This table lists the regulatory components considered in the Th cell differentiation network model, along with their qualifications, behaviours and related references.

Next, logical rules are defined for each regulatory component to specify its *target* activity level according to the levels of its regulators. Whereas some authors consider standard logical functions for all components, we do not impose such a restriction. For example, although STAT1 and IL21 both receive three activatory interactions, their logical rules differ: the rule assigned to STAT1 stipulates that STAT1 = 1 **if** IFNBR = 1 **or** IFNGR = 1 **or** IL27R = 1 (otherwise STAT1 = 0), whereas the rule assigned to IL21 stipulates that IL21 = 1 **if** NFAT = 1 **and** proliferation = 1 **and** STAT3 = 1.

Given a regulatory graph and starting from a (set of) initial state(s), successor states can be recursively computed. This results in a new graph, called *state transition graph*, which describes the dynamical behaviour of the system. In this graph, a node denotes a *state* of the system (*i.e.*, a tuple giving the levels of the regulatory components), whereas an arc linking two states denotes a possible *state transition*.

Considering a state, if the activity level of a component differs from the target level defined by its logical rule, there is an updating call on the corresponding variable. We generally assume that a state has as many successors as updated components (fully asynchronous dynamics), potentially leading to alternative behaviours. The computation of the state transition graph can be restricted by considering a set of priority classes. Each regulatory component is then associated to a priority class and is updated only in the absence of concurrent updating call with a higher priority [25].

Considering a state transition graph, it is particularly interesting to identify the *attractors*, which correspond to potential asymptotical behaviours, being either *stable states* (states without any successor) or *cyclical components* (denoting oscillatory behaviours).

### Model reduction

The asynchronous dynamical analysis of increasingly large regulatory graphs can be very challenging due to the exponential growth of the state transition graphs. A solution consists in reducing the model by removing (intermediate) components.

We have recently proposed an algorithm to automatically compute the logical rules for a user-defined reduced model [22]. Starting with a detailed model, the computation of a reduced model is performed by iteratively removing regulatory components. Auto-regulated components are not entitled for removal, thereby avoiding the loss of dynamical properties associated with regulatory circuits (see below). Removing a regulatory component G implies a revised wiring where all targets of G are directly regulated by the regulators of G. The logical rules of the targets of G are modified accordingly to conserve the (indirect) effects of its regulators.

This reduction method ensures the preservation of a number of dynamical properties of the original model. In particular, stable states and more complex attractors are conserved. However, additional cyclical attractors may arise from the isolation of transient cycles of the original system. An attractor which is reachable in the reduced model is also reachable in the full model, but the reverse is not always true, as the reduction may lead to the loss of some trajectories (see [22] for further details).

### Feedback circuit analysis

One asset of the logical framework is the possibility to analyse the dynamical roles of the *regulatory circuits* (circular chains of interactions) embedded in a regulatory graph. The dynamical roles of isolated regulatory circuits depend on their signs. A circuit is positive if it encompasses an even number of inhibitions, whereas a negative circuit involves an odd number of inhibitions. During the eighties, R. Thomas conjectured that a positive circuit is required to generate multiple attractors, whereas a negative circuit is necessary for sustained oscillations [26]. Since then, these rules have inspired several theorems in different mathematical frameworks (see [27] for a brief survey). In this work, we focus on positive circuits, which enable the existence of alternative differentiated cell lineages, each corresponding to one attractor.

While the presence of a positive circuit is necessary for the existence of multiple stable states, this condition is not sufficient. Indeed, when regulatory circuits are embedded in large networks, external regulators may prevent some circuits to generate the expected dynamical behaviour. Circuit functionality contexts can then be defined in terms of constraints on the values of the regulators of circuit members (see [28] for further details). In practice, in large regulatory graphs, a tiny fraction of the regulatory circuits are usually functional (*i.e.* have non-empty functionality contexts). Such circuit functionality analysis pinpoints crucial regulatory structures (usually with a predominance of short circuits) that are responsible for salient dynamical features.

### GINsim, a software dedicated to logical dynamical modelling

Developed in Java, the software *GINsim* provides a user interface to set up logical models, as well as to compute state transition graphs under diverse updating schemes, including the use of priority classes [29]. GINsim further implements an efficient algorithm for the determination of all stable states of a logical model, as well as the reduction method and the regulatory circuit analysis briefly mentioned above (see also [28]).

We used GINsim to build a comprehensive logical model by integrating a large set of documented molecular actors and interactions. The resulting model is available in the model repository linked to the GINsim website. As GINsim allows the user to document a model by associating annotations (textual comments, links and references) with each component, extensive documentation has been integrated in the model file (see Dataset S1).

### Model simulations

In the case of the model presented here, we have to deal with many input nodes that collectively handle local environmental conditions. Varying the values of these inputs, all possible fates of a given cell can be computed in terms of attractors reachable in these diverse local environments.

Our simulations aim at determining how naive cells can differentiate into specialised cells, depending on local environments, and how these differentiated cells respond to environmental changes. In this respect, we have defined a set of prototypic local environments (*i.e.* in terms of specific combinations of values for APC and external cytokines), that are biologically meaningful. For each of these environments, a simulation is performed starting from a state corresponding to naive Th cells. All the resulting attractors consist in stable states (*i.e.* attractors reduced to a unique state, each corresponding to a cell lineage). These stable states are in turn taken as initial states for a new round of simulations, considering different local environments. We have chained such simulations until no new stable state could be found, thereby allowing us to assess the behaviour of each cell lineage depending on the local environment. These simulations have been performed using priority classes assigning a higher rank to the receptors (TCR and cytokine receptors) and their downstream signal transducers and/or transcription factors (NFAT and the STATs) over other components.

### Stable states, expression patterns and cell types

As we wish to take into account the roles of various external cytokines on the activation and differentiation of Th cells, we expect to find many possible stable states, some differing from each other only by minor component activities, and thus corresponding to the same Th subtype. Similar stable states can be grouped and denoted by the value of a vector encompassing the major transcription factors (GATA-3, T-bet, ROR
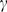
t, Foxp3) and cytokines expressed by specific Th subtypes (IFN-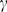, TGF-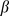, IL-2, etc.). The resulting *patterns* corresponding to different (quiescent, activated, or anergic) modalities of the same Th subtype can be further grouped into cell *constellations*, where simulated cellular state transitions are denoted by arcs linking the corresponding cell types.

## Results

### Building blocks of the T cell regulatory network

**Figure 1:**
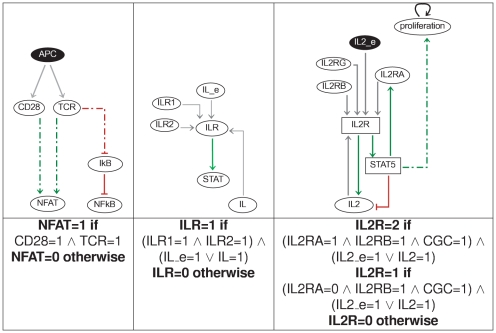
Model building blocks. **Left:** (simplified) TCR signalling pathway. The node denoted NFAT represents the joint activity of NFAT and AP1. **Middle:** generic cytokine module. IL_e represents the cytokine present in the environment; IL represents the autocrine production of the same cytokine; ILR1 and ILR2 represent two different receptor sub-chains; ILR represents the activated receptor, which in turn activates STAT. **Right:** IL2R regulation and its effect on cell proliferation. The bottom row gives the logical functions used for one of the components of each module. “
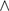
” and “
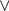
” stand for AND and OR logical operators, respectively.

The logical formalism enables a modular approach for the construction of large regulatory network models, where parts of the network are defined and studied separately, before merging them to generate a comprehensive model. The Th cell network has been constructed by combining several “modules” illustrated in Figure 1 and described below.

#### TCR signalling and costimulation

The first building block is the TCR signalling module involved in the control of all aspects of Th cell life cycle, including activation and differentiation. A comprehensive Boolean model of this signalling cascade has been recently published [30]. In brief, T cells receive coordinated inputs from the antigen presenting cell via the TCR itself (an heterodimer of an alpha chain with a beta chain) and via the costimulation receptor CD28. For the sake of simplicity, we represent these coordinated inputs by a single variable, denoted APC, and we assume that these signals converge downstream to activate NFAT/AP1 (represented by the component NFAT) and NFkB (see Figure 1, left). This simplified pathway captures the essential dynamical features of the more complete model network analysed in [30].

#### Cytokine signalling

Cytokines play an important role in the control of T cell differentiation. Cytokine signalling proceeds via the JAK-STAT pathway. Cytokine binding to its receptor chains leads to the phosphorylation of specific JAK and STAT factors, the latter being translocated into the nucleus where they activate the transcription of target genes.

A generic logical model for cytokine signalling is shown in Figure 1 (middle). The generic cytokine is represented by two components, IL and IL_e. IL represents the production of the cytokine by the T cell under study, whereas IL_e represents the presence of cytokine in the environment, to which Th cells (when activated) may contribute. The presence of cytokines in the environment (whatever their autocrine or paracrine origin) is considered in terms of initial conditions in the simulations.

The generic cytokine module has been replicated and adapted for different cytokines, taking into account the relevant receptor chains, and the specific JAK and STAT components downstream (see Table 2). ILR represents the activated state of the cytokine receptor: it is active when its subunits are crosslinked to the corresponding cytokine, which may have an autocrine or paracrine origin. This can be represented by the logical function ILR1 **and** ILR2 **and** (IL **or** IL_e). Note that several subchains and STATs are shared between different cytokine modules. We consider that a STAT is active when at least one of its activating cytokine receptors is active.

**Table 2:** List of cytokines.

Cytokines	Chains	Targets
IFNG	IFNGR1, IFNGR2	STAT1
IL4	IL4RA, CGC	STAT5, STAT6
IL6	IL6RA, GP130	STAT3
IL10	IL10RA,IL10RB	STAT3
IL12	IL12RB1, IL12RB2	STAT4
IL15	IL2RB, CGC, IL15RA	STAT5
IL21	CGC, GP130	STAT3
IL23	IL12RB1, GP130	STAT3
IL27	GP130, IL27RA	STAT1, STAT3

List of the cytokines considered in our model, each corresponding to an instance of the generic module shown in Figure 1 (middle). For each cytokine, the corresponding receptor sub-chains and downstream targets are specified. CGC stands for Common Gamma Chain. The IL-15 receptor has three subchains (versus two in the generic module), all of which are required for proper signalling.

To enable the analysis of the model in terms of stable states, we provisionally ignore SOCS-dependent negative feedbacks.

#### IL2 and cell cycle

**Figure 2:**
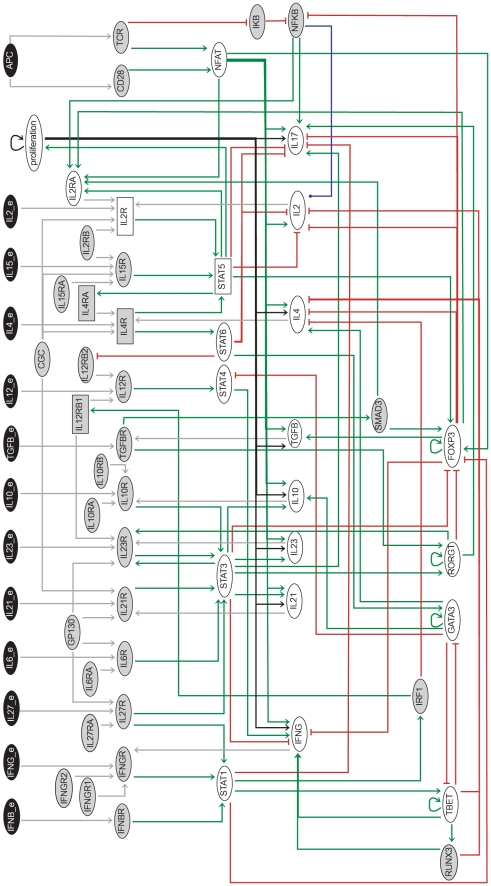
Th differentiation regulatory graph, encompassing 65 components. The 13 input components are colored in black. Ellipses denote Boolean components while rectangles denote ternary components. Green arrows denote activations, whereas red blunt ones denote inhibitions. A peculiar blue arrow denotes the unique dual interaction. The greyed-out components have been reduced to generate the regulatory graph displayed in Figure 3.

As IL2 plays a particular role in the control of Th cell differentiation and proliferation, it deserves a more detailed description. The IL2/IL2R module shown in Figure 1 (right) extends the general cytokine module by considering an explicit feedback onto IL2 through STAT5, as well as the effect of STAT5 on cell proliferation [31] (for details on the regulation of IL2 and of its receptor, see [32]). Note that, in our full model (Figure 2), IL2 and IL2RA are further regulated by NFAT, NFKB and FOXP3, not shown in Figure 1.

IL2 signalling is also involved in the activation of induced cell death (for a review, see [33]), which is not explicitly considered here.

### Regulatory network controlling Th cell activation and differentiation

All the modules must then be integrated to build a comprehensive model. The TCR signalling module functions as an input, since it is not regulated by any other component. Several cytokine receptors share subchains and targets. For example, the common gamma chain (CGC) is shared by IL2, IL4, IL7, IL9 and IL15 receptors that lead to the activation of STAT5 (cf. Table 2). Furthermore, the cytokine modules are connected through a number of cross-regulatory interactions. Figure 2 displays the whole regulatory graph assembled for this study, while Table 1 provides a brief description of all the components included in the model (Dataset S1). The logical rules associated with each component are given in Text S1, along with biological justifications and bibliographical references.

### Reduced model

Which cell types and differentiation pathways can be predicted from the logical model just described? The different cell types correspond to attractors in the state transition graph, *i.e.* regions of the state transition graph from which the system cannot escape. Among these attractors, stable states can be readily determined [28]. Other attractors may consist in (intertwined) terminal cycles. Their identification requires a thorough analysis of the state transition graph.

**Figure 3:**
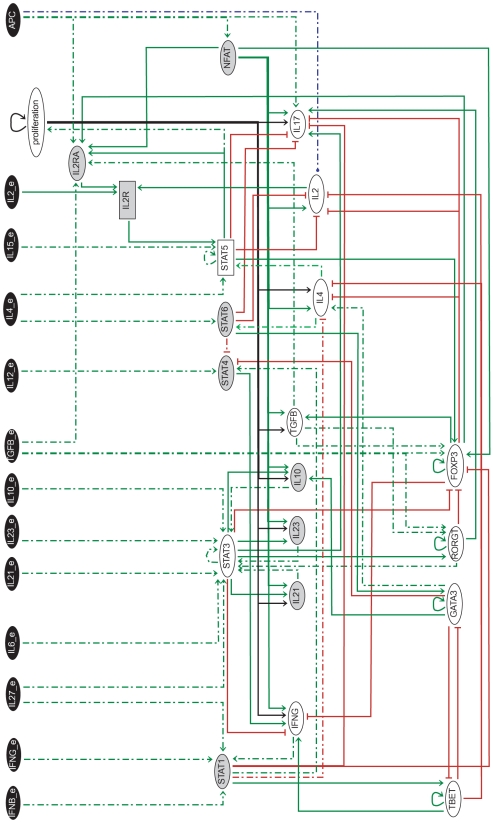
Reduced Th regulatory graph, encompassing 34 components. This graph has been obtained by applying the reduction method described in Section “Model reduction” to the full model shown in Figure 2. Indirect interactions resulting from the reduction are displayed using dotted lines. Greyed-out components can be further reduced to generate a more compact model, which still keeps the most relevant Th differentiation markers.

As our regulatory network encompasses too many components to enable a direct analysis of the full state transition graph, we have applied the reduction method described in Section “Model reduction”. Regarding the selection of the components for reduction, we face a compromise between computational performance and biological readability. Selecting TCR, CD28, cytokines receptors and their subchains, along with the intermediate components RUNX3, IRF1, SMAD3, IKB and NFKB for reduction, we obtain a regulatory graph containing 34 components (see Figure 3 and Dataset S2). Although the resulting logical model is still too large to compute the whole state transition graph (encompassing over 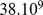 states), it is now amenable to simulations starting from selected initial states. Indeed, in this regulatory graph, 13 out of 34 components represent environmental conditions (inputs), which are fixed for each simulation. With 21 internal components, the system has “only” about 4.5 millions of possible states, most of which are not reachable when starting from a specific initial state.

**Figure 4:**
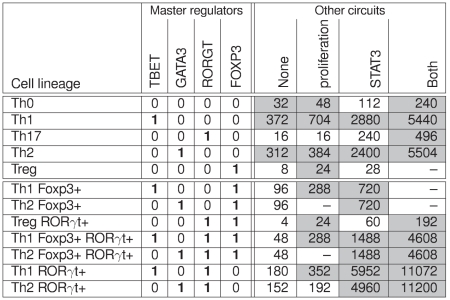
Definition of alternative Th subtypes based on the expression of the master regulators. Each of the four master genes considered (TBET, GATA3, RORGT and FOXP3) is positively auto-regulated. The first five rows correspond to the canonical Th cell subtypes expressing no (Th0) or a single master regulator (Th1, Th17, Th2, Treg). The remaining rows correspond to hybrid Th cell subtypes that express more than one of the master regulators, *i.e.* that show hybrid patterns. Additional positive circuits (proliferation and STAT3-related) generate further subtypes. The circuit analysis predicts 48 stable patterns (4 for each of the 12 groups; each pattern corresponds to one cell of the table under the heading “Other circuits”). Only 28 of these patterns (greyed cells) are compatible with at least one of the input combinations considered here (cf. Figure 5). The values in the cells indicate how many input combinations are compatible with this stable state. Five patterns are not compatible with any input combination (cells with dashes).

A further reduction leads to a graph conserving the 13 inputs, but only 12 internal nodes: TBET, GATA3, RORGT, FOXP3, STAT3, STAT5, IL2, IL4, IFNG, IL17, TGFB and proliferation. Using this compact model, we could compute the full state transition graphs for relevant input combinations. This led us to identify 28 context-dependent stable states (corresponding to the greyed-out cells in Figure 4) and to verify the absence of cyclic attractors for the polarising environments considered. Since our reduction preserves attractors [22], we can conclude that, for these environments, all attractors of the original model are indeed stable states. Furthermore, whenever a stable state can be reached in the compact model from specific initial conditions, we can conclude that it is also the case for the full model.

#### Regulatory circuits and cell fate decisions

Regulatory circuits are known to play a role in the emergence of essential dynamical properties (cf. Section “Feedback circuit analysis”). The regulatory graph presented in Figure 2 encompasses 612 circuits. We expect only a small fraction of them to be functional, with a predominance of short positive circuits. Of special interest are the auto-activations of the four master regulators: TBET, GATA3, FOXP3, and RORGT, along with the cross-inhibitory circuit involving TBET and GATA3. Indeed, each of these circuits may function as a switch to maintain the differentiation of a specific Th subtype.

Considering that each functional positive circuit can lead to two attractors, the four auto-activations can possibly generate 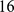 (*i.e.* 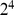) attractors (combining the two possible states of the four related components). However, the cross-inhibitory circuit (TBET-GATA3) ensures that TBET and GATA3 are mutually exclusive, leading to the 12 possible combinations (rows) shown in Figure 4. The first five patterns correspond to the expected canonical Th0, Th1, Th2, Th17 and Treg subtypes, while the last seven patterns correspond to non-canonical Th subtypes expressing more than one master regulator, *i.e.* with markers corresponding to more than one canonical subtype. Each of these stable states may be compatible or not with specific input configurations. Moreover, additional positive circuits may be functional and thereby introduce further expression variability. For example, the circuit involving STAT3 can lead to stable patterns differing by the value of STAT3 and its target cytokine genes.

**Figure 5:**
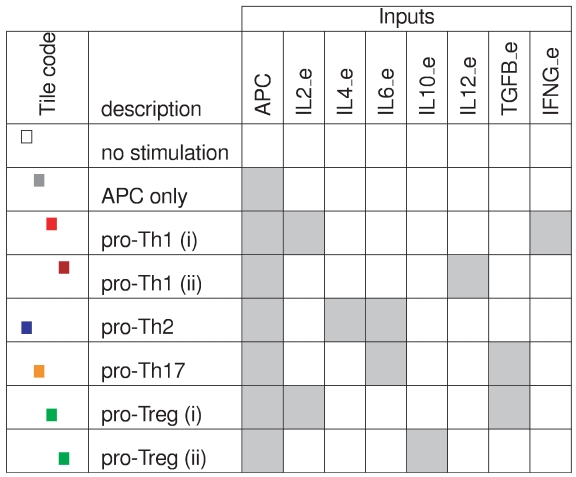
Environmental conditions used for the simulations. Each row corresponds to one prototypic environment, defined in terms of combinations of APC and of seven different cytokine inputs. Presence/absence of the different inputs is denoted by grey/white cells. The coloured tile code defined in the first column is used in Figure 7 to denote environmental conditions.

**Figure 6:**
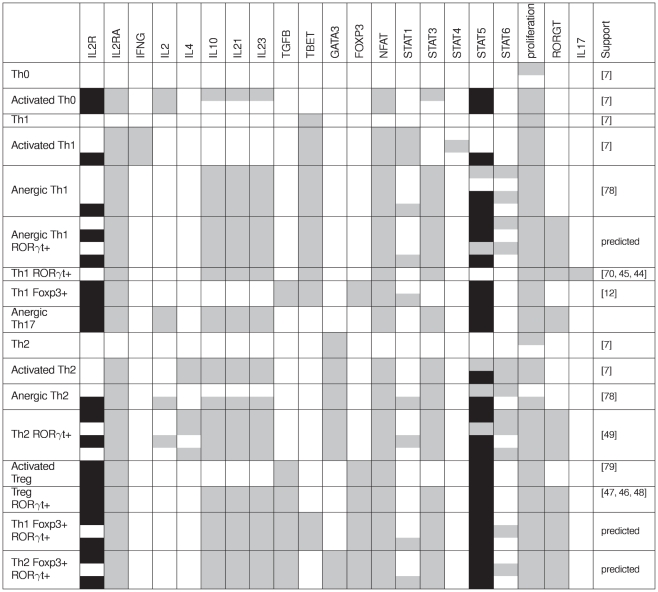
Context-dependent stable states and their component expression patterns. A grey cell denotes the activation of the corresponding component (column entries) for the corresponding stable state (row entries). Black cells denote higher activity levels (in the case of multi-level components). Note that the values of the input nodes are omitted here. A state stable for a given input combination may become unstable for other input values. Relationships between these stable states and selected environmental conditions (described in Figure 5) are given in Figure 7. Activated cells (*i.e.* expressing NFAT and producing lineage-specific cytokines) and anergic cells (*i.e.* expressing NFAT but no lineage-specific cytokine) are indicated, when this classification clearly applies. Note that different stable states sharing a common pattern in terms of expression of master regulators but differing in the expression of other components are identified as the same Th cell subtype (as in the case of Th2 Foxp3+ ROR
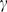
t+ subtype at the end of the table).

To analyse how cell fate depends on initial and environmental conditions, we have iterated several round of simulations, using the reduced model shown in Figure 3 (34 components) and the prototypic environments described in Figure 5 (cf. Section “Model simulations”). In the first round of simulations, we have used an initial state corresponding to the Th0 subtype. Consequently, we have obtained the stable states listed in Figure 6 and associated with specific cell types (first column) according to the expression of key lineage and activity markers.

#### Plasticity of Th cell types

**Figure 7:**
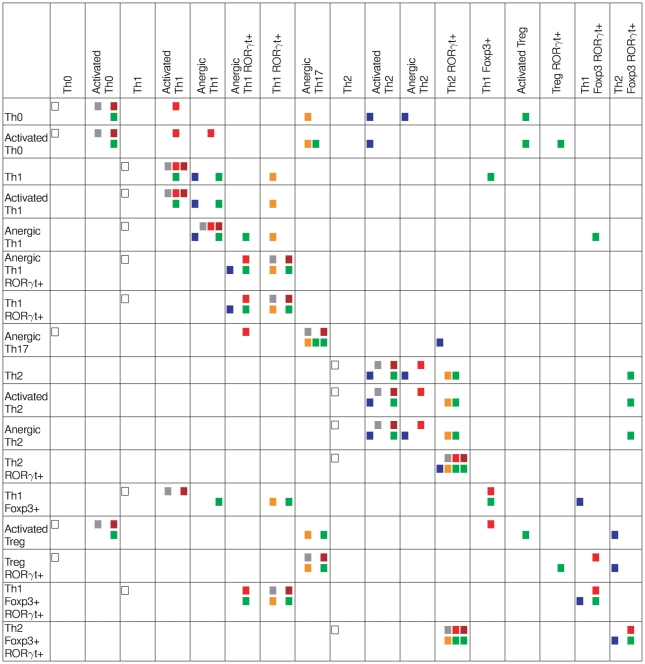
Stability of Th cell subtypes and environment-dependent transitions. This figure summarises several simulation rounds, displaying the context-dependent stable states (column entries) reached depending on eliciting initial states (row entries) and environmental conditions (coloured tiles). The coloured tile code for environmental conditions is defined in Figure 5.

**Figure 8:**
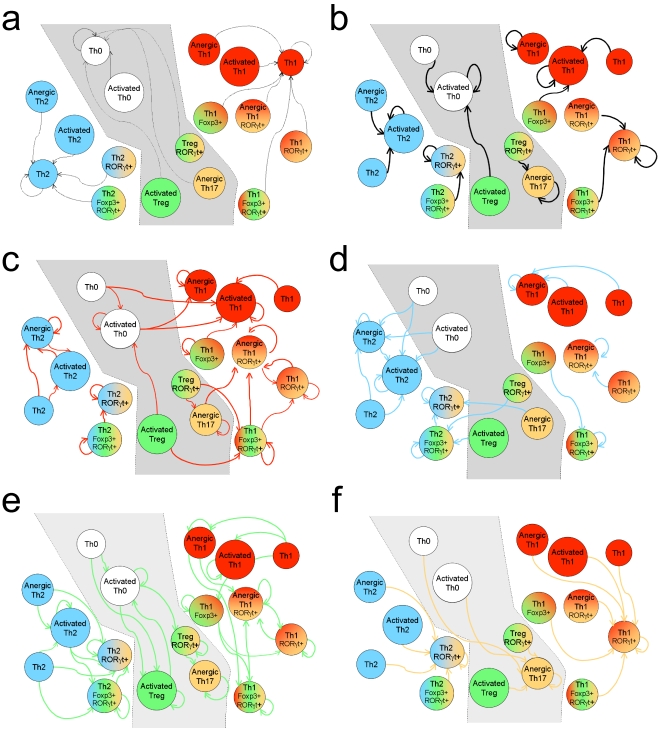
Graphical representation of the plasticity of cell subtypes depending on the environment. The Th cell subtypes observed *in silico* are grouped into three main *constellations* (Th0, Th1 and Th2, delimited by different backgrounds). The different panels correspond to different environmental conditions listed in Figure 5: (**a**) no stimulation, (**b**) APC only, (**c**) pro-Th1, (**d**) pro-Th2, (**e**) pro-Treg, and (**f**) pro-Th17. Arrows between cell lineages denote switches elicited by the corresponding environment. Cell colouring denotes the activity of the master regulators: GATA3 (blue), T-bet (red), Foxp3 (green) and ROR
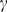
t (orange).

To check the stability of the identified Th cell subtypes in changing environments, we have performed a series of simulations using each of the stable states listed in Figure 6 as initial configuration with each of the prototypic environments listed in Figure 5. The results are summarised in Figures 7 and 8.

In the absence of any input from the environment, resting Th0, Th1, and Th2 are the only three attractors. These three cell types can thus be considered as reference states, to which the other stable states can be associated depending on the expression of characteristic markers. However, these groups or *constellations* of states are not disconnected. Obviously, any cell turning into a Th0 subtype can readily switch to states belonging to either of the two other constellations, depending on the polarising environment. According to our simulations, using proper (sequences of) environmental input conditions, several differentiated Th subtypes could be *reprogrammed* into various other subtypes.

By and large, Th1 cells tend to remain within the Th1 state constellation, which includes activated, resting, and anergic variants. For example, stimulation by APC will activate resting Th1 into activated Th1 cells, inclusively in a pro-Treg environment. In either a pro-Th2 or pro-Treg environment, Th1 cells will become anergic, while they will tend to express ROR
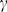
t with or without IL-17 in a pro-Th17 environment. Anergic Th1 cells, as well as non-canonical Th1 and Th17 mixed cell types, will remain so provided that APC stimulus is sustained, but most likely turn to resting Th1 cells in the absence of sustained TCR signalling. In a pro-Treg environment, resting Th1 cells can up-regulate Foxp3 and further express both T-bet and Foxp3. This chimeric cell type can loose Foxp3 under several conditions: in absence of TCR stimulus and of other stimuli, they will revert to the Th1 resting state; in the presence of APC alone (without pro-Treg stimuli) or together with IL-12 (pro-Th1), they turn into the canonical activated Th1 state; whereas in the presence of IL-12 they turn into an anergic Th1 state.

Th2 differentiated cells expressing GATA-3 are even more robust than Th1 cells and will always remain within the Th2 constellation (denoted in blue in Figure 8). In pro-Th1 environments, all Th2-like cells converge towards the anergic Th2 subtype, devoid of cytokine production. According to our model, in pro-Th17 or pro-Treg environments, Th2 cells may turn on ROR
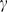
t and Foxp3, respectively. While Foxp3 expression will be lost in the absence of IL-2, the expression of ROR
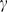
t is more robust and can be maintained in all environments eliciting TCR activation.

A subset of Th17 cells expressing ROR
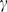
t overlaps with all other lineages. This results from the positive auto-regulation of ROR
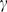
t, which is barely affected by other transcription factors. Thus, an environment rich in TGF-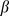 and IL-6 can lead to the activation of ROR
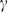
t in virtually any cell type (Figure 8). This expression will be stable until the cell switches to a resting state in the absence of TCR signals from the APC. Interestingly, the production of IL-17 by Th17 cells expressing ROR
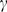
t alone is always transient, while a hybrid Th1 ROR
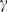
t+ cell is predicted to express this cytokine stably.

Treg cells can be maintained only in the presence of TCR-stimulus delivered by APC and of IL-2 produced by other T cells. However, in strong polarising environments, Tregs may differentiate into mixed cell types associated with the Th1 or Th2 cell constellations. For example, ROR
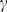
t could be activated in Th1 Foxp3+ cells dwelt in a pro-Th2 environment. Similarly, ROR
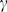
t could be activated in canonical Foxp3+ cells dwelt in a pro-Th1 environment. These cells can then loose Foxp3 expression in several environmental conditions, from pro-regulatory to pro-inflammatory environments, coming closer to the Th1 subtype. However, Foxp3 expression might be maintained in other experimental conditions, in particular in sustained pro-Th2 environments. Foxp3+, ROR
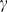
t+, and GATA-3+ cells can arise from anergic Th17 cells or from several Foxp3+ precursors by a sustained pro-Th2 environment. Finally, Th0 cells can potentially turn into Th2 via regulatory or Th17 intermediates.

## Discussion

We have reconstructed the molecular network controlling the activation and differentiation of Th cells and asked how many stable states to expect, considering that these cells face a changing local environment during their life span.

Components and cross-regulatory links were extracted from the literature and, in some cases, from previous logical models [18], [34]. It is likely that components or links were missed. To this adds the problem of the definition of the logical functions driving the behaviour of the components, particularly those involving complex regulatory mechanisms.

This cautionary remark notwithstanding, our current model recapitulates the differentiation of naive cells into Th1, Th2, Th17 and Treg subtypes. Strikingly, our model also gives rise to hybrid states expressing markers characteristic of two or more canonical cell types. Cyclic attractors (potentially corresponding to oscillatory behaviour) have been found only in restricted environmental situations, whose biological relevance remains to be assessed. Furthermore, our model analysis emphasises an unexpected plasticity of the canonical cell types. Indeed, according to the model, both Foxp3+ regulatory T cells and Th17 cells are highly plastic and labile, whereas Th1 and Th2 subtypes are more readily maintained across different environmental conditions.

Based on our results, canonical Foxp3+ regulatory T cells would not be truly lineage committed, but would rather correspond to a context-dependent stable state of the underlying regulatory network. This is surprising considering the fundamental role of these cells in avoiding autoimmunity. According to our model analysis, the maintenance of a regulatory T cell phenotype would require sustained TCR/CD28 and IL-2 signals. Indeed, the absence of TCR stimulation can lead to the loss of Foxp3 expression and a conversion into a Th0 phenotype. Depending on inputs, these cells can regain Foxp3 expression but also differentiate into other cell types. Our model further predicts that Treg cells may differentiate into Th1 or Th2 subtypes in proper polarising environments. This plasticity of regulatory Foxp3+ T cells is supported by several recent reports [13], [35], [36] showing that fluorescence sorted Foxp3+ T cells loose Foxp3 expression and their suppressive capacity, under experimental conditions consistent with our simulations (*e.g.* loss of Foxp3 expression after adoptive transfer of regulatory T cells in the absence of conventional Foxp3- CD4 T cells, which provide IL-2 to the former [13]). However, in addition to cells with labile Foxp3 expression, the regulatory Foxp3+ T pool apparently also contains irreversibly committed cells, perhaps of thymic origin [35], which cannot be easily explained with our model.

In our model analysis, Treg lability clearly depends on the assumption that Foxp3 expression requires Stat5 and AP-1/NFAT transcriptional activities. This might turn out to be an oversimplification as we might have overlooked some mechanisms preventing regulatory cells to become conventional T cells and vice-versa. Some data suggest that locus epigenetic regulation is needed for sustainable Foxp3 expression [37], and that regulatory miRNAs are likely involved [38]. Alternatively, the irreversible regulatory T cell state might be explained by an additional positive loop involving a transcription factor upstream of Foxp3. According to this scenario, Foxp3 could merely control anergy and suppressive functions of regulatory T cells, but would not be the Treg master regulator. The recent characterisation of Th cells with partial Treg phenotype in Foxp3-deficient mice supports this scenario [39]. Yet another alternative is that higher order regulatory mechanisms stemming from intercellular interactions and population dynamics may stabilise the Treg phenotype. Indeed, differential equation models [40] suggest that stable regulatory T cells pools could be maintained despite a continuous interconversion of cells. Further evidence is provided by loss or gain of Treg phenotype depending on the remaining populations [13].

Now, if our prediction of pervasive T cell plasticity is correct, how can the classes of immune responses in which they predominate be so robust? This point is particularly intriguing in the context of natural tolerance, presumably depending on labile Tregs, or yet regarding persistent memory responses to bacterial infections. Here again, the stability of the responses could stem from regulatory feedbacks at the level of the T cell populations, as intercellular interactions and cell population dynamics might sustain and stabilise specific combinations of Th subtypes. Regarding Treg mediated tolerance, stable and robust coexistence of conventional Th cells and regulatory T cells has been obtained using a cross-regulatory model encompassing positive and negative feedback circuits at the population level in [41]. In stationary populations, the production of IL-2 by conventional T cells sustains the expression of Foxp3 by Tregs. Disruption of this balance can lead to the conversion of Tregs into conventional Th phenotypes, as observed in adoptive transfers [13]. Regarding Th cell memory, the recent finding that memory cells relocate to the bone marrow, where they remain quiescent in special niches [42], raises the possibility that stromal cells may provide the environment necessary to sustain a context-dependent memory state.

Among the model stable states, those corresponding to hybrid cell types expressing two or more Th master regulators are particularly striking. Most of these hybrid cell types co-express ROR
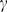
t with another Th master regulator. The remaining hybrids combine the expression of Foxp3 with other master regulators. The generation of these hybrid stable states might reveal missing cross-inhibitory mechanisms between ROR
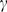
t and Foxp3, or yet among these and the two other master regulators. The introduction of such cross-inhibitory circuits would grant mutually exclusive expression of master regulators (as assumed for GATA-3 and T-bet and generalised in [21]). Some indications of additional mutual inhibitions among master regulators can be found in the recent literature (*e.g.* between Foxp3 and ROR
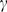
t [37], [43]). Alternatively, since predicted hybrid cell types tend to be anergic (*i.e.* do not produce cytokines), they could have been overlooked in routine experimental assays characterising cell populations based on cytokine expression. In fact, the existence of some of these hybrid phenotypes is supported by recent studies relying on more sophisticated quantitative assays, thereby suggesting that mutually exclusive master regulator expression might not be a general principle of the Th regulatory network architecture. For example, hybrid cells expressing both IFN-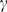 and IL-17, dependent on T-bet and ROR
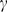
t respectively, have been observed by flow cytometry [44] or clonal analysis [45], thus resembling our *in silico* Th1 ROR
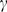
t+ subtype (row 7 in Figure 6). Other reports point to the generation of hybrid cells similar to our Treg ROR
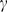
t+ subtype (row 15 in Figure 6), following the induction of ROR
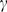
t and IL-17 expression in regulatory Foxp3+ T cells, both in mice [46], [47] and humans [48]. Furthermore, in the presence of IFN-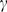, Foxp3+ regulatory T cells have been shown to up-regulate T-bet [12], leading to hybrid cells akin our Th1 Foxp3+ subtype (row 8 in Figure 6). Finally, genetically enforcing GATA-3 expression does not prevent differentiation of Th17 cells, leading to GATA-3+ IL-17 expressing hybrid cells [49]. Whether these cells are an artefact or reflect naturally occurring activated Th2 ROR
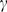
t+ cells (row 13 in Figure 6) remains to be established.

Altogether, these observations support the existence of several of our predicted environment-dependent Th subtypes, thereby warranting the investigation of the other predicted hybrids (*e.g.* Th1 Foxp3+ ROR
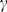
t+ or Th2 Foxp3+ ROR
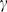
t+). Indeed, we have identified several environment-dependent Foxp3+ subtypes expressing other master transcription factors characteristic of canonical Th1, Th2 or Th17 cells. What are the functional implications of these chimeric regulatory cells? Since Foxp3 inhibits the expression of the cytokines downstream of GATA-3, T-bet, or ROR
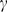
t, these hybrid cells are expected to be anergic. In an environment dominated by other effector Th cells, up-regulation of a subset of genes not suppressed by Foxp3 could allow chimeric cells to mingle within effector T cells, profiting from local growth or survival factors, as suggested by population dynamical modelling [41]. This hypothesis is supported by the recent observation that essential Th1 or Th2 regulatory factors are required in Foxp3+ cells to enable proper control of Th1 or Th2 responses, respectively [12], [50].

The number of reported cytokines grows rapidly. Tentatively, some of these cytokines could be predominantly expressed by specific, yet undiscovered, cell types. One might thus wonder what part of our conclusions could be retained as our knowledge on the Th cell differentiation network grows. In this respect, we have extended earlier models for Th1/Th2 bipolarisation [18], [34], with the aim to account for two additional cell fates (Treg and Th17 subtypes). Interestingly, although our model accounts for many more cell fates, Th0, Th1 and Th2 subtypes are maintained as reference subtypes, around which are organised the cellular constellations displayed in Figure 8. This organisation can be related to the definition of a robust GATA-3 and T-bet cross-regulatory module [51] progressively embedded in a more comprehensive regulatory network.

However, even this well established paradigm has been recently challenged by reports demonstrating simultaneous and sustained GATA-3 and T-bet co-expression in reprogrammed Th2 cells [52]. Although mechanistic details still need to be worked out, this finding suggests that Th cell plasticity may be even greater than fostered by our model. Further refinements of the model (*e.g.* the introduction of an additional level for T-bet, and potentially of additional components) will be required to properly model the generation of such hybrid Th2+1 cells.

More generally, the characterisation of the roles of additional cytokines, transcription factors, and regulatory RNAs, or yet the delineation of epigenetic regulatory mechanisms, should enable proper model extensions and refinements, thereby leading to refined predictions, while preserving the functional roles of the most salient regulatory modules.

In conclusion, our results indicate that the pool of CD4 Th cells is highly heterogeneous and that the structure of the gene network promotes this heterogeneity. Diversity of antigen receptors and their crossreactivity are the most salient features of the adaptive immune system of the vertebrates, allowing the host to recognize and potentially react to numerous antigens. The heterogeneity and plasticity of Th cell types emphasised here might further contribute to the capacity of the immune system to deal with wide contingencies. Arguably, such plasticity does not fit well with the classical depiction of T cell differentiation potential in terms of a branching tree. Instead, our computational study points to a reticulate network of alternative, environment-dependent, differentiation and reprogramming events.

## Supporting Information

Dataset S1 Complete annotated Th model. Compressed archive (with extension ZGINML) for the complete, annotated Th differentiation model, including the model file (XML file with extension GINML) and parameter files. This file can be directly opened with the freely available GINsim software.
(0.01 MB ZIP)

Dataset S2 Reduced Th differentiation model. Compressed archive (with extension ZGINML) for the reduced Th differentiation model, including the model file (XML file with extension GINML) and parameter files. This file can be directly opened with the freely available GINsim software.
(0.01 MB ZIP)

Text S1 Th differentiation model documentation. Documentation for the complete Th differentiation model,which include supporting data and links to relevant databases for each model component.
(0.18 MB PDF)
