# Supplementary material for: Diversity and Plasticity of Th Cell Types Predicted from Regulatory Network Modelling
Source: PLoS Comput Biol. 2010 Sep 2;6(9):e1000912. doi: 10.1371/journal.pcbi.1000912 (PMC2932677; doi:10.1371/journal.pcbi.1000912)
Supplement: Text S1 — Th differentiation model documentation. Documentation for the complete Th differentiation model,which include supporting data and links to relevant databases for each model component. (0.18 MB PDF) [file pcbi.1000912.s003.pdf]

Description of the model

"Th_differentiation_full_annotated_model_26May2010"

This logical model integrates the documented cross-regulations between key transcription factors and the relevant signalling pathways controlling Th cell differentiation.
Using a reduction method and other analysis tools, we could show that this model generate specific stable states corresponding to canonical Th1, Th2, Th17 and Treg subtypes, which coexist with other transient hybrid cell types that co-express combinations of Th1, Th2, Treg and Th17 markers in an environment-dependent fashion.
The dynamical analysis of this model thereby provides novel insights into the heterogeneity and plasticity of late Th cell lineages.
Furthermore, this model enables *in silico* investigation of the nature of Th subtypes, as well as of potentially novel differentiation or reprogrammation pathways.

Node ID	Val	Logical function	Comment
APC		Input component	Antigen Presenting Cell.<br>This input node denotes proper presentation of an antigen recognised by the Th TCR.
IFNB_e		Input component	● hugo:5434<br>External source of interferon-beta.
IFNG_e		Input component	● hugo:5438<br>External source of interferon-gamma.
IL2_e		Input component	● hugo:6001<br>External source of interleukine-2.
IL4_e		Input component	● hugo:6014<br>External source of interleukine-4.
IL6_e		Input component	● hugo:6018<br>External source of interleukine-6.
IL10_e		Input component	● hugo:5962<br>External source of interleukine-10.
IL12_e		Input component	● hugo:5969<br>● hugo:5970<br>External source of interleukine-12.
IL15_e		Input component	● hugo:5977<br>External source of interleukine-15.
IL21_e		Input component	● hugo:6005<br>External source of interleukine-21.
IL23_e		Input component	● hugo:15488<br>External source of interleukine-23.
IL27_e		Input component	● hugo:19157<br>External source of interleukine-27.
TGFB_e		Input component	● hugo:11766<br>External source of Transforming Growth Factor beta.
CD28	1	APC	● hugo:1653<br>CD28 provides a co-stimulatory signal to the TCR, required for the activation of Th cells.
IFNBR	1	IFNB_e	● hugo:5432<br>Upon ligand binding, the IFN-beta receptor activates STAT1.
IFNGR	1	IFNGR1 & IFNGR2 & (IFNG | IFNG_e)	The IFN-gamma receptor requires the IFNGR1 and IFNGR2 subchains and activates STAT1 upon binding.

IL2R	1	CGC & IL2RB & !IL2RA & (IL2 | IL2_e)	● ref:Kim06<br>The IL-2 receptor requires the CGC and IL2RB subchains, and activates STAT5 upon binding.<br>The IL2RA subchain is needed for the high affinity response.
	2	CGC & IL2RB & IL2RA & (IL2 | IL2_e)	
IL4R	1	CGC & IL4RA:1 & (IL4 | IL4_e)	The IL-4 receptor requires the CGC and IL4RA subchains and activates STAT6 and STAT5 upon binding.
	2	CGC & IL4RA:2 & (IL4 | IL4_e)	
IL6R	1	GP130 & IL6RA & IL6_e	The IL-6 receptor requires the IL6RA and GP130 subchains and activates STAT3 upon binding.
IL10R	1	IL10RA & IL10RB & (IL10 | IL10_e)	The IL-10 receptor requires the IL10RA and IL10RB subchains, and activates STAT3 upon binding.
IL12R	1	IL12RB1 & IL12RB2 & IL12_e	● ref:Kano2007<br>The IL-12 receptor requires the IL12RB1 (at high level) and IL12RB2 subchains and activates STAT4 upon binding.
IL15R	1	CGC & IL15RA & IL2RB & IL15_e	The IL-15 receptor requires the CGC, IL15RA and IL2RB subchains and activates STAT5 upon binding.
IL21R	1	GP130 & CGC & (IL21 | IL21_e)	● hugo:6006<br>The IL-21 receptor requires the GP130 and CGC subchains, and activates STAT3 upon binding.
IL23R	1	GP130 & IL12RB1 & (IL23 | IL23_e) & STAT3 & RORGT	● hugo:19100<br>● ref:Ivanov07<br>The IL-23 receptor requires the GP130 and IL12RB1 subchains and activates STAT3 upon binding.<br>According to (Ivanov et al, 2007), STAT3 and RORgt are needed for its expression (most likely for the expression of its subchains).
IL27R	1	GP130 & IL27RA & IL27_e	The IL-27 receptor requires the IL27RA and GP130 subchains and activates STAT1 and STAT3 upon binding.
TCR	1	APC	T Cell Receptor, activated by antigen presentation.
TGFBR	1	TGFB | TGFB_e	Upon ligand binding, the TGF-beta receptor activates SMAD3.
IFNGR1	1	(basal value)	● hugo:5439<br>IFNGR1 is a subchain of the IFN-gamma receptor.
IFNGR2	1	(basal value)	● hugo:5440<br>IFNGR2 is a subchain of the IFN-gamma receptor.
GP130	1	(basal value)	● hugo:6021<br>Glycoprotein 130 (IL-6 Signal Transducer) forms a complex with binded receptor of the IL-6 familly.<br>It is required for signal transduction of these receptors (IL6R, IL21R, IL23R and IL27R in this model).
IL6RA	1	(basal value)	● hugo:6019<br>IL-6 receptor alpha is a subchain of the IL-6 receptor.
IL12RB1	1	(basal value)	● hugo:5971<br>● ref:Kano2007<br>IL-12 receptor beta 1 is a subchain of the IL-12 and IL-23 receptors. According to Kano et al (2007), IRF1 promotes higher level of IL12RB1, which is required for IL-12 signalling, but not for IL-23.
	2	IRF1	
CGC	1	(basal value)	The Common Gamma Chain (IL2RG), is shared by several cytokine receptors (IL2R, IL4R, IL15R and IL21R).

IL12RB2	1	(basal value)	● hugo:5972<br>● ref:Mendoza06<br>● ref:Szabo1997b<br>IL-12 receptor beta 2 is a subchain of the IL-12 receptor.<br>According to (Szabo et al, 1997), STAT6 blocks the IL12 pathway by inhibiting IL12RB2.
IL10RB	1	(basal value)	● hugo:5965<br>IL10 receptor beta is a subchain of the IL-10 receptor.
IL10RA	1	(basal value)	● hugo:5964<br>IL10 receptor alpha is a subchain of the IL-10 receptor.
IL4RA	1	!STAT5:2	● hugo:6015<br>● http://www.ncbi.nlm.nih.gov/pubmed/18820682<br>IL-4 receptor alpha is a subchain of the IL-4 receptor.<br>IL-2 upregulates IL4RA expression in a STAT5 dependent way and thereby promotes augmented IL-4RA expression and priming for responsiveness to IL4 (LIao et al, 2008).
	2	STAT5:2	
IL15RA	1	(basal value)	● hugo:5978<br>IL-15 receptor alpha is a subchain of the IL-15 receptor.
IL2RB	1	(basal value)	● hugo:6009<br>IL-2 receptor beta is a subchain of the IL-2 and IL-15 receptors.
IL2RA	1	(SMAD3 | FOXP3 | STAT5 | NFKB) & NFAT	● hugo:6008<br>● ref:Kim06<br>IL-2 receptor alpha (CD25) is the high affinity subchain of the IL-2 receptor.<br>According to (Kim et al, 2006), it is activated by NFAT, SMAD3, NFKB and STAT5.<br>It is also activated by FOXP3 in CD4+CD25+ Treg.<br>Its presence enables higher activation of the IL-2 receptor.
IL27RA	1	(basal value)	● hugo:17290<br>IL-27 receptor alpha is a subchain of the IL-27 receptor.
IFNG	1	proliferation & !FOXP3 & !STAT3 & NFAT & ((TBET & RUNX3) | STAT4)	● hugo:5438<br>● ref:Djuretic2007<br>● ref:Bettelli2005<br>● ref:Mendoza06<br>The gamma interferon is produced by active Th1 cells.<br>Its production requires NFAT (blocked by FOXP3), cell proliferation, and either IL12 (through STAT4) or Tbet (in cooperation with RUNX3).<br>It is inhibited by STAT3.

IL2	1	((NFAT & !FOXP3) | NFKB) & !(STAT5 & STAT6) & !(NFKB & TBET)	● hugo:6001<br>● ref:Villarino2007<br>● ref:Rudensky2006<br>● ref:Hwang2005<br>● ref:Kim06<br>● http://www.ncbi.nlm.nih.gov/pubmed/12646638<br>Interleukine 2 is one of the most actively studied cytokines (for a review, see Kim et al, 2006).<br>Its production requires NFAT (blocked by FOXP3) but not proliferation (unlike assumed for other cytokines).<br>According to (Villarino et al, 2007), STAT5 (activated by IL2/4/15) and STAT6 (activated by IL4) cooperate to inhibit IL2 production.<br>RelA has been shown to induce IL-2 expression following transient overexpression (Rao et al, 2003).<br>According to (Hwang et al, 2005), Tbet cooperates with relA (a NFkB subunits to inhibit IL2.<br>Hence, the interaction from NFKB onto IL2 has a context-dependent sign.
IL4	1	NFAT & proliferation & GATA3 & !FOXP3 & !((TBET & RUNX3) | IRF1)	● hugo:6014<br>● ref:Djuretic2007<br>● ref:Bettelli2005<br>● ref:Elser02<br>IL4 is produced by Th2 cells.<br>Its production requires NFAT (blocked by FOXP3, see Bettelli et al, 2005), proliferation and GATA3.<br>it is inhibited by Tbet (cooperating with RUNX3, see Djuretic et al, 2007) and IRF1 (Elser et al, 2002).
IL10	1	(GATA3 | STAT3) & NFAT & proliferation	● hugo:5962<br>● ref:McGeachy07<br>● ref:Mendoza06a<br>Interleukine-10 is activated by GATA3 and STAT3.<br>As for other cytokines, its production requires NFAT and proliferation.
IL21	1	NFAT & proliferation & STAT3	● hugo:6005<br>● ref:Korn07<br>● ref:Zhou07<br>● ref:Brenne2002<br>Interleukines 21 and 23 are sequentially activated by IL6 through STAT3 (see Zhou et al, 2007).<br>As for other cytokines, their production requires NFAT and proliferation.
IL23	1	NFAT & proliferation & STAT3	● hugo:15488<br>● ref:Zhou07<br>Interleukines 21 and 23 are sequentially activated by IL6 through STAT3 (see Zhou et al, 2007).<br>As for other cytokines, their production requires NFAT and proliferation.
TGFB	1	NFAT & proliferation & FOXP3	● hugo:11766<br>TGF-beta is believed to be important in regulation of the immune system by Treg cells and apparently block the activation of lymphocytes and some phagocytes.<br>The mechanism leading to its expression in Treg cells is unknown.<br>Here, we assume an activation by FOXP3.

TBET	1	(TBET | STAT1) & !GATA3	● hugo:11599<br>● ref:Mendoza06<br>T-bet (TBX21) is the main transcription factor of the Th1 lineage.<br>It is activated by STAT1, self-maintained and inhibited by GATA3.
GATA3	1	(GATA3 | STAT6) & !TBET	● hugo:4172<br>● ref:Mendoza06<br>GATA3 is the main transcription factor of the Th2 lineage.<br>It is activated by STAT6, self-maintained and inhibited by Tbet.
FOXP3	1	STAT5 & NFAT & (FOXP3 | (SMAD3 & !STAT1 & !(STAT3 & RORGT))	● hugo:6106<br>● ref:Pyzik07<br>● ref:Floess2007<br>● ref:Yao2007<br>● ref:Tone2008<br>Foxp3 is the main known marker of regulatory T cells.<br>It is activated by NFAT, TGF-beta (through Smad3, see Tone et al, 2008) and STAT5 (see Yao et al, 2007).<br>It is inhibited by STAT1 (binding site observed in Floess et al, 2007), STAT3 (Yao et al, 2007) and RORgt.<br>We asumme that Foxp3 can maintain its own expression (binding sites observed in Floess et al, 2007) in presence of NFAT and STAT5.<br>We also assume that STAT3 and RORgt cooperate to inhibit Foxp3.
NFAT	1	CD28 & TCR	● hugo:7775<br>● hugo:7776<br>● hugo:7777<br>● hugo:7778<br>The Nuclear Factor of Activated T cells (NFAT) familly of transcription factors encompass 5 members.<br>The nucleus localisation of NFATc1 to NFATc4 depends on the presence of calcineurin (and thus of the TCR activation).<br>This node represents the presence of NFAT (and AP1) in the nucleus.
STAT1	1	IFNBR | IFNGR | IL27R	● hugo:11362<br>● ref:Mendoza06<br>● ref:Weaver07<br>● ref:Kamiya2004<br>STAT1 is activated by IL-27, IFN-beta and IFN-gamma receptors.
STAT3	1	IL6R | IL10R | IL21R | IL23R | IL27R	● hugo:11364<br>● ref:Brenne2002<br>● ref:Mendoza06a<br>● ref:Weaver07<br>STAT3 is activated by IL-6, IL-21, IL-23, IL-27 and IL-10 receptors.
STAT4	1	IL12R & !GATA3	● hugo:11365<br>● ref:Mendoza06a<br>STAT4 is activated by IL-12 receptor and inhibited by GATA3.<br>Mendoza et al (2006) proposed it as intermediate in IFN-gamma inhibition by GATA3.
STAT5	1	!IL2R:2 & !IL4R:2 & (IL4R:1 | IL2R:1 | IL15R)	● hugo:11366<br>● hugo:11367<br>● ref:Kim06<br>STAT5 is activated by IL-4, IL-15 and IL-2 receptors.<br>A high level of IL2R promotes a high level of STAT5, required to activate cell proliferation.
	2	IL2R:2 | IL4R:2	

STAT6	1	IL4R	● hugo:11368<br>● ref:Mendoza06<br>STAT6 is activated by IL-4 receptor.
SMAD3	1	TGFBR	● hugo:6769<br>● ref:Kim06<br>SMAD3 is activated by TGF- beta receptor and involved in the activation of IL2RA and FOXP3.
IRF1	1	STAT1	● hugo:6116<br>● ref:Kano2007<br>IRF1 is required for the activation of IL12RB1 and for the inhibition of IL4 by STAT1.
RUNX3	1	TBET	● hugo:10473<br>● ref:Djuretic2007<br>RUNX3 is activated by T-bet, with which it cooperates to activate IFN-gama and inhibit IL-4.
proliferation	1	STAT5:2 | proliferation	● ref:Moriggl99<br>● ref:Bird98<br>This component denotes that the cell proliferation has been activated.<br>It is used as a switch required for the expression of most cytokines.<br>It is activated by IL2 through STAT5 (see Moriggl et al, 1999).
NFKB	1	!IKB & !FOXP3	● ref:Bettelli2005<br>● http://www.ncbi.nlm.nih.gov/pubmed/12646638<br>NF-kappa B is inhibited by I-kappa B and Foxp3 (according to Bettelli et al, 2005).<br>The NF-kB family is composed is composed of NF-kB1, NF-kB2, RelA, RelB and c-Rel. c-Rel has been solidly implicated in the regulation of IL-2 transcription by chromatin remodelling accross the IL-2 gene (Rao et al, 2003). Other components such as RelA were shown to induce IL-2 expression following transient overexpression.<br>Hence, the interaction from NFKB onto IL2 has a context-dependent sign.
IKB	1	!TCR	The NF-kappa B inhibitor I-kappa B is inhibited by the TCR signal.
RORGT	1	(TGFBR & STAT3) | (RORGT & (TGFBR | STAT3))	● ref:Zhou07<br>● ref:Manel2008<br>RORGT is a key actor in Th17 differentiation.<br>According to (Zhou et al, 2007), RORgt is activated by TGFb + STAT3 (activated by interleukines 6, 21 and 23).<br>Here, we assume that RORgt is self-regulated (as the other master regulators).
IL17	1	NFAT & proliferation & RORGT & !FOXP3 &<br>NFKB & STAT3 &<br>!(STAT5 | STAT1 | STAT6)	● hugo:5981<br>● ref:Zhou2008<br>IL17 cytokines are produced by TGFb+IL6 induced Th17 cells when stimulated by IL23 and (IL18 or IL1b).<br>STAT3 and RORGT activate IL17 cooperatively.<br>The action of RORGT can be blocked by FOXP3 (Zhou et al, 2008). STAT3 and NFkB are required to activate IL17.<br>IL2 inhibits IL17 through STAT5. STAT1, STAT5 and STAT6 can bind on the IL17 promoter.<br>An inhibitory effect for Tbet and IL4 has also been reported.<br>Coexpression of IL17 and IFNg has been observed, indicating that these inhibitions may be overcomed.

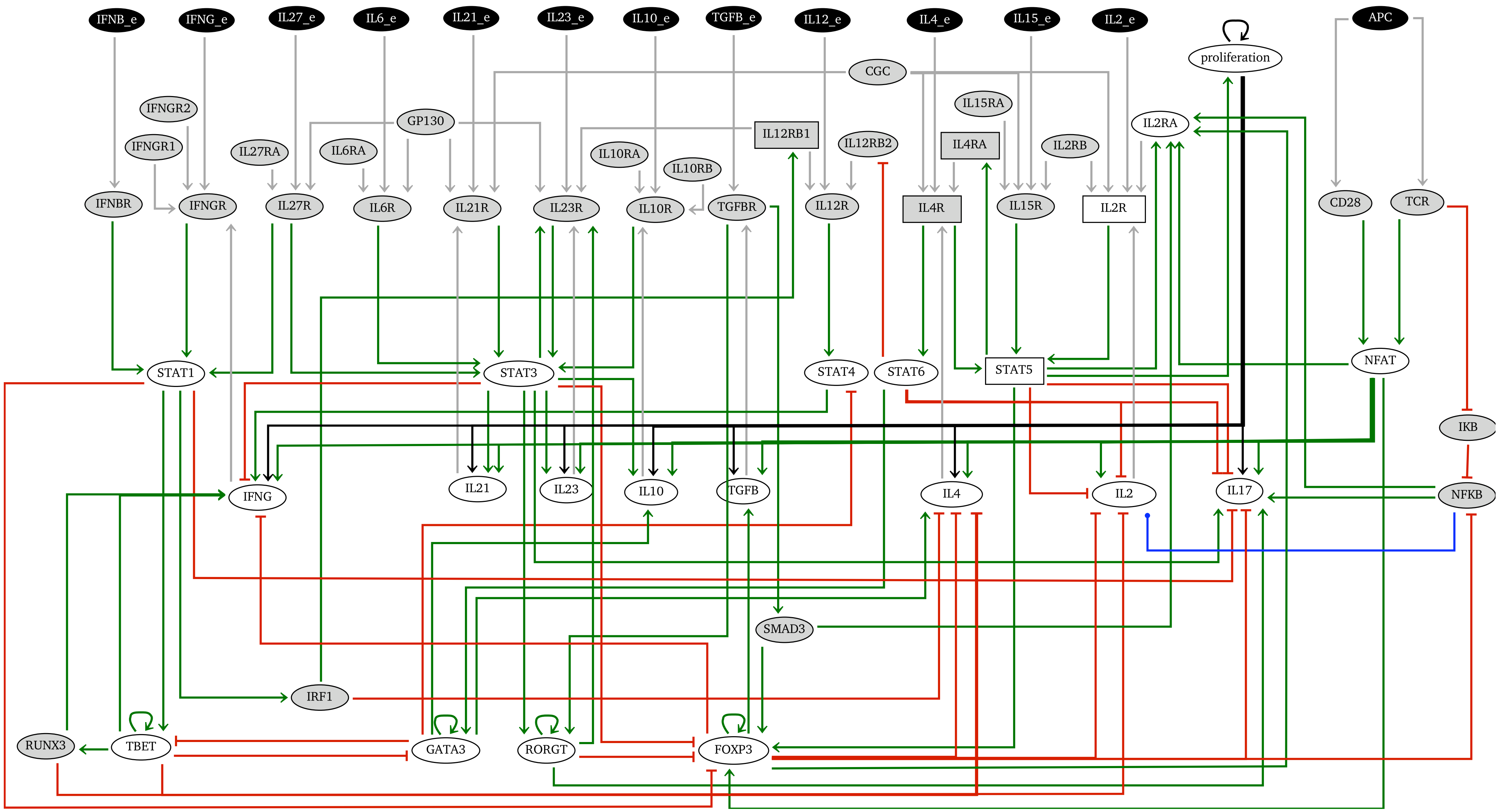
IFNB_e
IFNG_e
IL27_e
IL6_e
IL21_e
IL23_e
IL10_e
TGFB_e
IL12_e
IL4_e
IL15_e
IL2_e
APC
proliferation
CGC
IFNGR2
GP130
IL15RA
IL2RA
IFNGR1
IL27RA
IL6RA
IL10RA
IL12RB1
IL12RB2
IL4RA
IL2RB
IL10RB
IFNBR
IFNGR
IL27R
IL6R
IL21R
IL23R
IL10R
TGFBR
IL12R
IL4R
IL15R
IL2R
CD28
TCR
STAT1
STAT3
STAT4
STAT6
STAT5
NFAT
IKB
IFNG
IL21
IL23
IL10
TGFB
IL4
IL2
IL17
NFKB
SMAD3
IRF1
RUNX3
TBET
GATA3
RORGT
FOXP3
